# Psychogenic Nonepileptic Seizures Associated with an Eating Disorder and PTSD Are Responsive to Cognitive Processing Therapy

**DOI:** 10.1155/2023/5539951

**Published:** 2023-11-20

**Authors:** Marina G. Gearhart, Timothy D. Brewerton

**Affiliations:** ^1^Monte Nido and Affiliates, Miami, FL, USA; ^2^Department of Psychiatry and Behavioral Sciences, Medical University of South Carolina, Charleston, SC, USA

## Abstract

**Objective:**

Eating disorders (EDs) are often associated with prior histories of trauma, subsequent PTSD and related psychiatric comorbidities. There is a paucity of information about their relationship to somatic symptom disorders, specifically psychogenic nonepileptic seizures (PNES), a type of functional neurological symptom disorder or conversion disorder.

**Methods:**

We report a case of a 39-year-old bisexual female with bulimia nervosa (BN), PTSD, recurrent major depressive disorder (MDD), cannabis use disorder, and PNES who responded to integrated trauma-focused treatment during residential ED treatment using cognitive processing therapy (CPT). Symptoms of ED, PTSD, major depression, and state-trait anxiety were measured using validated assessment instruments.

**Results:**

During the course of CPT treatment, the patient's total scores on the PTSD Symptom Checklist for DSM-5 (PCL-5) went from 59 to 26, which is below the diagnostic threshold for PTSD. In addition, she demonstrated improvements in the Eating Disorder Examination Questionnaire (EDE-Q) Global Severity score, the Eating Disorder Inventory (EDI-2) total score, the Patient Health Questionnaire (PHQ-9) total score, the Spielberger State and Trait Anxiety Inventory scores, and the Eating Disorder Quality of Life (EDQOL) total score. Furthermore, her PNES also abated, and she remained seizure free for ∼1 year following discharge with the exception of one short seizure, per report of the patient.

**Conclusion:**

The use of CPT as part of an integrated trauma-informed treatment approach during residential ED treatment was successful in a woman with PNES, BN, PTSD, MDD, and cannabis use disorder.

## 1. Introduction

Eating disorders (EDs) are known to be commonly associated with a variety of comorbid psychiatric disorders [1, 2]. Prior histories of trauma and the development of posttraumatic stress disorder (PTSD) and related disorders in the context of EDs have been well-established in the scientific literature [3–10]. Furthermore, integrated treatment approaches that include trauma-focused treatments have emerged as viable treatments for these complex multimorbidities [11–15].

Somatic symptom disorders (SSDs) (previously known as somatoform disorders) are trauma-related conditions that have also been found to be associated with EDs [16, 17]. The related phenomenon of somatic dissociation has also been found to be associated with EDs [18, 19]. Psychogenic nonepileptic seizure disorder (PNESD), a subtype of functional neurological symptom disorder (FNSD) or conversion disorder, is classified in DSM-5 as a SSD [20]. PNESD is associated with somatic dissociation and high degrees of psychiatric comorbidity, trauma, and PTSD [21–23]. However, there is a paucity of information about the association of PNESD with EDs [24, 25].

The recommended treatment of PNESD involves a coordinated, multidisciplinary, holistic approach with a strong psychotherapeutic, cognitive–behavioral basis, which is also utilized in the treatment of EDs [26–31]. The identification and treatment of underlying PTSD and other comorbidities may be a central component of treatment and recovery from PNESD with PTSD, yet there is a lack of information about the use of trauma-focused approaches for this highly incapacitating combination of disorders, which exhibit greater severity and worse physical health than PNES without PTSD [32]. Prolonged exposure (PE) therapy has demonstrated efficacy in the treatment of patients with dually diagnosed PNES and PTSD [33], but we could find no reports of the treatment of PNES comorbid with PTSD and EDs. Cognitive processing therapy (CPT) is an evidence-based specific form of cognitive behavioral therapy (CBT) that has been shown to be effective in decreasing the symptoms of PTSD [34–36]. CPT is as clinically efficacious as PE but has a lower dropout rate than PE [36]. Recent research has also shown that CPT may be effectively incorporated into an overall treatment program for ED patients with PTSD, which has been adopted by Monte Nido and Affiliates programs as the preferred treatment approach for traumatized patients [11, 37, 38]. CPT is typically delivered over 12 sessions and assists patients in learning how to identify, challenge and modify maladaptive cognitions related to their trauma. The three primary principles of CPT include: (1) avoiding avoidance, (2) identifying stuck points, and (3) using Socratic questioning [34]. These principles were emphasized as essential clinical approaches that extended beyond the confines of CPT alone. Given the above, we therefore report our experience with an integrated trauma-focused treatment approach in residential treatment (RT) using CPT for PTSD in a woman with PNES and an ED.

## 2. Case Report

The patient is a 39-year-old white bisexual woman who presented to a residential ED facility for treatment of her ED, PTSD, depression, suicidal ideation, cannabis use, panic attacks, fear of the dark, and shaking episodes of unknown etiology. Initial evaluation revealed the following diagnoses: (1) bulimia nervosa (BN), (2) major depressive disorder (MDD), recurrent, unspecified, (3) anxiety disorder, unspecified, (4) cannabis use disorder, (5) PTSD, (6) slow transit constipation, (7) gastro-esophageal reflux disease (GERD) without esophagitis, (8) nausea, (9) restless legs syndrome, and (10) sphincter of Oddi dysfunction. The diagnoses of slow transit constipation, GERD without esophagitis, nausea, restless legs syndrome, and sphincter of Oddi dysfunction were diagnosed by the patient's outpatient gastroenterologist prior to ADM. The gastrointestinal (GI) related disorders caused some physical pain for the patient while restoring weight and managing nutrition needed for ED recovery. The patient's pain was managed with continuation of medication prescribed by her GI doctor. The patient's stay was 84 days in total.

Her ADM medications included the following:

1. quetiapine 25 mg as needed for anxiety, 100 mg at bedtime for insomnia, and 50 mg twice daily as needed for anxiety;
2. estradiol 3 mg/day status post total hysterectomy;
3. valacyclovir 500 mg/day for cold sores;
4. trazadone 25 mg at bedtime for insomnia;
5. gabapentin 800 mg twice daily and 200 mg at bedtime for anxiety;
6. escitalopram 30 mg/day for depression;
7. hyoscyamine 0.125 mg every 4 hr as needed for cramping/bloating;
8. polyethylene glycol 3350 17 g oral powder twice daily for constipation;
9. famotidine 20 mg daily for GERD;
10. omeprazole 20 mg daily as needed for heartburn;
11. cetirizine 10 mg daily for allergies.

The aforementioned medications were all prescribed by the patient's outpatient team, initially remained in place, but were then modified as assessment and treatment progressed by the on-site psychiatrist.

The patient's presenting problems were intense depression with suicidal ideation (SI), history of substance use (mixed amphetamine salts, alcohol), cannabis use disorder, several traumatic episodes, including sexual, and physical assaults, during adulthood, increasing “seizures”, binge/purge cycle, phobia of leaving her house in the dark or being home alone that lead to difficulty in work, and separation from husband during stay. Patient arrived to RT with a low sense of self and high engagement in behaviors. She had two suicide attempts prior to ADM; one was at age of 15 years when she consumed a bottle of fluoxetine capsules, and her second was at age 28 year when she took bottle of lorazepam. While in treatment, she received three times weekly individual psychotherapy sessions, multiple group sessions daily, a weekly session with a dietitian, a weekly session with the psychiatrist, and patient and therapist engaged in walks in the dark for exposure. The exposure walks, at first, induced anxiety and often led to “seizures” (pseudoseizures). Her first episode during RT occurred ∼2 weeks after ADM, at which time she was transferred to the emergency room for evaluation. She reported that her “seizures” first began7-months before ADM to RT and had happened only a few times. After ADM these episodes gradually became more intense and increased in duration, particularly around stressful discussions, activities, and meal times. The patient reported that her family members were unclear what was occurring and that they assumed her symptoms were due to panic attacks. The patient believed her jerking movements were potentially related to Tourette's disorder. Patient estimated that she had 30–40 episodes between the first episode and through treatment in RT. Each episode lasted ∼20–30 min. She reports being attacked by a dog and ongoing sexual assault prior to the onset of her PNES, which began within 3 months following these traumatic episodes. She reported no history of head injury or adverse reaction to medications.

During her stay, she experienced an increase in “seizures” that included two visits to the emergency department (ED) to rule out ictal seizure activity. She received a head CT scan and EKG which were within normal limits, and the lab panel was unremarkable. The patient was seen by a neurologist who observed her having an episode in real time and ruled out ictal seizures or the need for an electroencephalogram (EEG) based on their semiology and the fact that there was no loss of consciousness. Three days later the patient was brought to the ED for a second time with myoclonic rhythmic jerking of her upper and lower extremity, truncal areas as well as head. She was able to follow movement tasks prompted by the provider and then tremors resumed at the end of the task. She was also able to speak during each of the episodes. The patient was diagnosed at that time with PNES, which she experienced as a relief and gave her hope for the recovery. The neurologist also noted that her PNES could be linked to a past history of trauma or PTSD, and this “clicked” for the patient and led her to become more receptive to receiving trauma-focused treatment. For the episodes that occurred during RT, the patient was also instructed by the psychiatrist to use ice packs to stimulate the vagus nerve, augment the parasympathetic nervous system, and reduce stress [39]. The patient's therapist (MG), guided patient through slow, direct movements such as opening and closing the fists and flexing and pointing the feet. The intention was to bring control to areas of the body not impacted by PNES. The therapist used techniques supported by training in Dance/Movement Therapy [40]. The patient reported that this helped to reduce anxiety of the unfamiliar experience of the PNES.

After the diagnoses of PNES and PTSD were established, the therapist recommended she begin CPT. Patient attributed the increase in psychogenic seizure activity to being in treatment where she did not have access to her maladaptive coping skills such as binge eating, purging, cannabis use, and other forms of cognitive avoidance. Patient was motivated to begin trauma work as she believed her seizures and all of her psychiatric disorders were directly linked to her past traumas. Patient did not experience PNES during CPT sessions; however, a few occurred following difficult sessions and while completing worksheets assigned for CPT. CPT involves writing an initial impact statement as to why one believes their most distressing trauma has occurred, which allows for patients to explore beliefs developed as a result of the trauma. Following writing the initial impact statement, therapist and patient work together to derive a list of stuck points, or cognitive distortions to be challenged related to the themes of safety, power/control, esteem, intimacy, and trust [34]. There was a noticeable shift in the language of the patient of the present study's initial impact statement and final impact statement written at the end of the 12 sessions. The initial impact statement included beliefs such as: “I can't protect myself;” “The world is unsafe;” “I am to blame for what has happened to me;” and “I am unworthy because of who I am.”. The final impact statement included “I learned that these behaviors are results of trauma that happened TO ME, not that I caused or created. I learned that my brain was made with a limited threshold for these traumatic events, and I learned that I have been over-medicated for a decade because my brain was doing a hell of a job repressing the heavy stuff and I didn't know the real issues. It was easier and safer to not feel. I then learned how to face it. How to recognize it and how to process it. This came in the form of PNES, flash back images, raw honesty with memories, trust in the process, and a lot of tears” (personal communication, June 25, 2022). Patient also reflected in her final impact statement, “I am exploring how I view safety—with the goal of challenging the hypervigilance and intrusive negative narrative. I am recognizing I am able to trust myself to make decisions and to set boundaries for myself. I matter. My voice matters. My emotions and feelings matter. My happiness matters and is worth living for. I am more than the trauma that has happened to me”. The qualitative data show the positive shift in mindset and ability to challenge cognitive distortions that derive from trauma. Patient can recognize the impact CPT work had on her somatic symptoms, PNES, as well.

Her motoric symptoms and anxiety decreased, and patient was able to complete walks on her own at night. Patient completed 12 sessions of CPT where she challenged negative core beliefs associated with trust, safety, power/control, intimacy, and esteem. Patient also attributes her progress to discontinuing or reducing the dosage of several medications (i.e., quetiapine lowered and moved from standing to as needed, trazadone lowered, and moved from standing to as needed, gabapentin discontinued and escitalopram discontinued), abstaining from Cannabis use, trauma work through CPT sessions and proper nourishment.

Medications upon DC included:

1. lorazepam 1 mg twice daily as needed for PNES/anxiety;
2. diphenhydramine 50 mg every 8 hr as needed for PNES/jerking symptoms, which was prescribed by the emergency room physician and was not found to be effective by the patient;
3. calcium carbonate 1,000 mg as needed for heartburn or nausea;
4. cetirizine 10 mg daily for allergies;
5. docusate sodium 100 mg twice a day for constipation;
6. digestive enzymes 1 capsule three times daily for sphincter of Oddi dysfunction;
7. estradiol 3 mg daily S/P hysterectomy;
8. famotidine 20 mg daily as needed for GERD;
9. hyoscyamine 0.125 mg every 4 hr as needed for cramping/bloating;
10. polyethylene glycol 3350 17 g oral powder twice daily for constipation;
11. natural calm 325 powder twice daily as needed for restless legs and constipation;
12. nitroglycerin 0.3 mg daily as needed for sphincter of Oddi spasm;
13. omeprazole 20 mg daily as needed for heartburn;
14. desvenlafaxine 100 mg daily for depression;
15. quetiapine 25 mg daily at bedtime for sleep, 25 mg twice daily as needed for anxiety, 50 mg daily at bedtime for insomnia;
16. trazadone 25 mg at bedtime as needed for insomnia;
17. ondansetron 4 mg every 8 hr as needed for nausea.

### 2.1. Assessments

**Figure 1:**
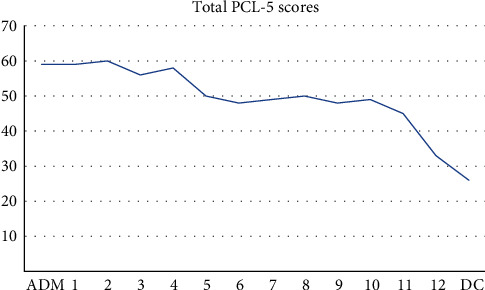
PTSD Symptom Checklist for DSM-5 (PCL-5) total scores during course of residential treatment from admission (ADM) to discharge (DC) with session numbers 1–12 in between.

The PTSD Symptom Checklist for DSM-5 (PCL-5) was used to measure PTSD symptoms at admission (ADM), prior to each session of CPT, and prior to discharge (DC) [41]. The Spielberger State Trait Anxiety Inventory (STAI) was used as a measure of state and trait anxiety [42] and the Patient Health Questionnaire (PHQ-9) was used as a measure of symptoms of major depression [43]. Additionally, the Eating Disorder Symptoms Questionnaire (EDE-Q) [44], Eating Disorder Inventory (EDI-2) [45], and the Eating Disorder Quality of Life Scale (EDQOL) [46] (Table 1) were administered. The aforementioned assessments were administered shortly after ADM and prior to DC. These assessments, apart from the weekly PCL-5, are not administered at other times during the stay, only at ADM and DC due to the protocol of the research department. Total scores on the PTSD Symptom Checklist for DSM-5 (PCL-5) over the course of treatment are shown in Figure 1, while the total scores on the STAI-S, the STAI-T, and the PHQ-9 at ADM and DC are shown in Table 1. Symptoms of PTSD, major depression and state–trait anxiety are noted to show improvement over the course of treatment. This research was approved by the Salus Institutional Review Board, and the patient in this report gave written informed consent for the use of her assessment results.

### 2.2. Followup

Per report of the patient 10 days after DC, she was 30-days seizure free, and at a year from ADM, patient states she continues to be seizure free apart from one following being witness to a car accident. Upon DC, her medication intake decreased significantly. She was discharged home with cetirizine, digestive enzymes, polyethylene glycol, docusate, omeprazole, natural calm, desvenlafaxine, quetiapine, estradiol, valacyclovir, trazadone as needed, lorazepam as needed for PNES symptoms, and hyoscyamine. She reports in an email 1-year post DC that she is happily divorced, working full-time, remains binge and purge free, has had no panic attacks or episodes of PNES, is able to drive and be outside at night, and is in a healthy new relationship with good communication and boundaries. She shares, “I've said it before, and I'll keep saying it. My time at residential/working with you, saved my life. It gave me a new life.”

## 3. Discussion

Prior research has not explored the connection of PNES as a response to trauma in those with EDs. The present case report demonstrates the successful use of CPT, an evidence-based trauma-focused therapy, as a successful intervention for treatment of a patient with PNES and BN. Not only did the patient experience a clinically significant decrease in PTSD symptoms and the cessation of psychogenic seizures, she had significant improvements in overall quality of life, major depression symptoms and state–trait anxiety symptoms from ADM to DC (Table 1). CPT is the form of integrated trauma therapy, the clinicians use with patients presenting to RT with PTSD diagnosis. CPT was developed specifically for the treatment of PTSD in both veteran and civilian populations and has been found in several controlled trials to be effective [34–36, 47]. CPT also significantly reduces comorbid symptoms of depression, disordered eating, dissociation, guilt, and borderline personality disorder, and enhances general mental health, quality of life, and social functioning [48–53]. CPT has been used by the clinicians for an array of trauma-related disorders and their symptoms and was beneficial in the treatment of this case of PNES.

## 4. Conclusion

A 39-year-old woman presented to a RT facility with BN, major depressive disorder, anxiety disorder, Cannabis use disorder, PTSD, extensive GI disorders and distress, and a history of suicidal ideation and attempts. Two weeks into the residential stay, she began experiencing seizure like symptoms that were eventually diagnosed by an ED neurologist as PNES. The treatment recommended was to address her trauma and underlying trauma-related psychiatric disorders. The therapist and patient completed 12 sessions of CPT in addition to her intensive individual and group therapy. As indicated by her PCL-5 scores, there was a reduction in PTSD symptomology (Figure 1) and a cessation of PNES symptoms. The patient attributes the improvement to her overall therapeutic work, and CPT in particular. In an email 1 year after DC, the patient writes, “I re-read my CPT first and last assignment and shed more than a few tears. They were tears of sadness for who I believed I was but also tears of joy and relief for having changed the wiring of my brain to see who I actually am.” This patient not only saw improvement in PNES and PTSD symptoms but found value in her life with decreased depression (Table 1), decreased anxiety (Table 1), increased self-esteem, and improved quality of life (Table 1). The literature has shown that CPT is effective in the treatment of PTSD, and the present study shows the treatment of PNES and comorbid ED (BN) are responsive to CPT delivered within an integrated, trauma-focused program.

## Figures and Tables

**Table 1:** Patient's admission and discharge scores.

	Admission	Discharge
EDEQ global		
Score	5.25	2.91
EDQOL		
Total score	2.36	1
PHQ-9		
Total score	19	7
STAI-S		
Score	63	40
STAI-T		
Score	74	43

*Note*. EDEQ = Eating Disorder Examination Questionnaire; EDQOL = Eating Disorders Quality of Life Scale; PHQ-9 = Patient Health Questionnaire; STAI-S = Spielberger State Trait Anxiety—State Anxiety score; STAI-T = Spielberger State Trait Anxiety—Trait Anxiety Score.

## Data Availability

The data do not come from publicly archived datasets. The data are owned by Monte Nido and Affiliates Research Department.

## Additional Points

*Limitations*. This is a single case report, and our findings cannot be generalized to all patients with PNES. A series of ED patients with PTSD and PNES is ideally needed in order to study the effectiveness of CPT as a form of treatment in this population. Although the present study lacks data about depression, anxiety, and ED symptomology throughout the stay, PCL-5 scores tend to be highly correlated to these measures [7, 54, 55]. Nevertheless, future prospective research would benefit from assessing the aforementioned throughout the course of treatment. In addition, further studies on the prevalence of PNES in ED populations would be informative, although somatoform symptoms have been reported in association with the individuals with EDs, particularly those with traumatic histories [16, 18, 19, 25, 56, 57].
